# Supplementary material for: Comparison of Functional Components and Antioxidant Activity of Lycium barbarum L. Fruits from Different Regions in China
Source: Molecules. 2019 Jun 14;24(12):2228. doi: 10.3390/molecules24122228 (PMC6632000; doi:10.3390/molecules24122228)
Supplement: Supplementary file 1 [file molecules-24-02228-s001.pdf]

Supplementary Materials:

Comparison of Functional Components and Antioxidant Activity of *Lycium barbarum* L. Fruits from Different Regions in China

Youyuan Lu [1], Sheng Guo [1,*], Fang Zhang [1], Hui Yan [1], Da-wei Qian [1], Han-qing Wang [2], Ling Jin [3], and Jin-ao duan [1,*]

[1] Jiangsu Collaborative Innovation Center of Chinese Medicinal Resources Industrialization, State Administration of Traditional Chinese Medicine Key Laboratory of Chinese Medicinal Resources Recycling Utilization, National and Local Collaborative Engineer Center of Chinese Medicinal Resources Industrialization and Formulae Innovative Medicine, Nanjing University of Chinese Medicine, Nanjing 210023, China ; luyylhy@163.com (Y. L.); fangzhang@njucm.edu.cn (F. Z.); glory-yan@163.com (H. Y.); qiandwnj@126.com (D. Q)

[2] School of Pharmacy, Ningxia Medical University, Yinchuan 750021, China; wwwhhq@163.com

[3] Gansu University of Chinese Medicine; zyxyjl@163.com

* Correspondence: guosheng@njucm.edu.cn (S. G.); dja@njucm.edu.cn (J.-A. D.); Tel.: +86-25-8581-1916 (S.G.); +86-25-8581-1291 (J.-A. D.)

1 Supplementary Methods (Analysis of individual phenolic compounds by HPLC)

1.1 Extract Preparation for Analysis of Individual Phenolic Compounds

The sample from NXZW (No. 5) was used to extract the individual phenolic compounds according to the method described by Zhang, et al. (Food Chemistry: 10.1016/j.foodchem.2016.01.046).

1.2 Analysis of Individual Phenolic Compounds by HPLC-DAD

HPLC-DAD analysis was performed on a Waters Alliance 2695 system (Waters, USA) consisting of a 2695 module and a 2998 DAD detector. Chromatographic separation was performed using a reverse phase column (Thermo Hypersil ODS-C18, 250 × 4.6 mm). According to the literature reported by Zhang, et al. (Food Chemistry: 10.1016/j.foodchem.2016.01.046), the mobile phase was composed of A (0.1% formic acid, aqueous) and B (methanol). Gradient elution was performed as follows: 0–20 min, 37–50% B; 20–35 min, 50–80% B; 35–40 min, 80–100% B; 40–50 min, 100% B; 50–60 min, 37–50% B. The column temperature was maintained at 25 °C and the flow rate was 0.7 mL/min. The detection wavelengths were set at 367 nm to detect flavonoids, 280 and 320 nm to detect phenolic acids.

2 Supplementary Figures

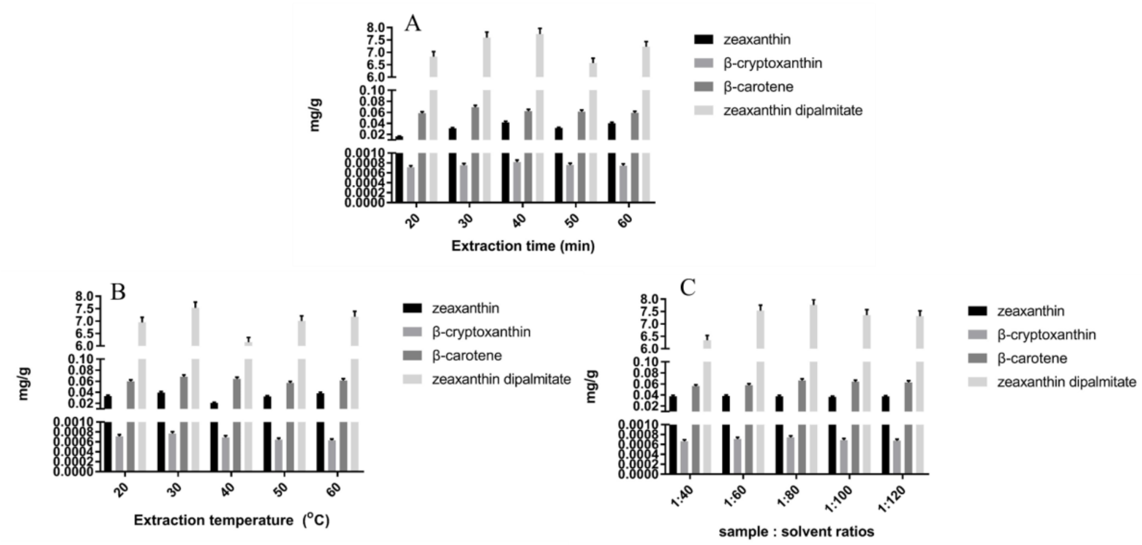

Figure S1. Optimization of samples solutions preparation for the carotenoids determination

A: different extraction times (the fruit powder (0.5 g) were extracted with 40 ml of hexane/acetone/ethanol (2:1:1, v/v/v) for 20, 30, 40, 50 and 60 min, respectively, at 30 °C using ultrasonic bath;

B: different extraction temperatures (the fruit powder (0.5 g) were extracted with 40 ml of hexane/acetone/ethanol (2:1:1, v/v/v) for 40 min at 20, 30, 40, 50 and 60 °C, respectively, using ultrasonic bath;

C: different sample : solvent ratios (the fruit powder (0.5 g) were extracted with 20, 30, 40, 50 and 60 ml of hexane/acetone/ethanol (2:1:1, v/v/v), respectively, for 40 min at 30 °C using ultrasonic bath).

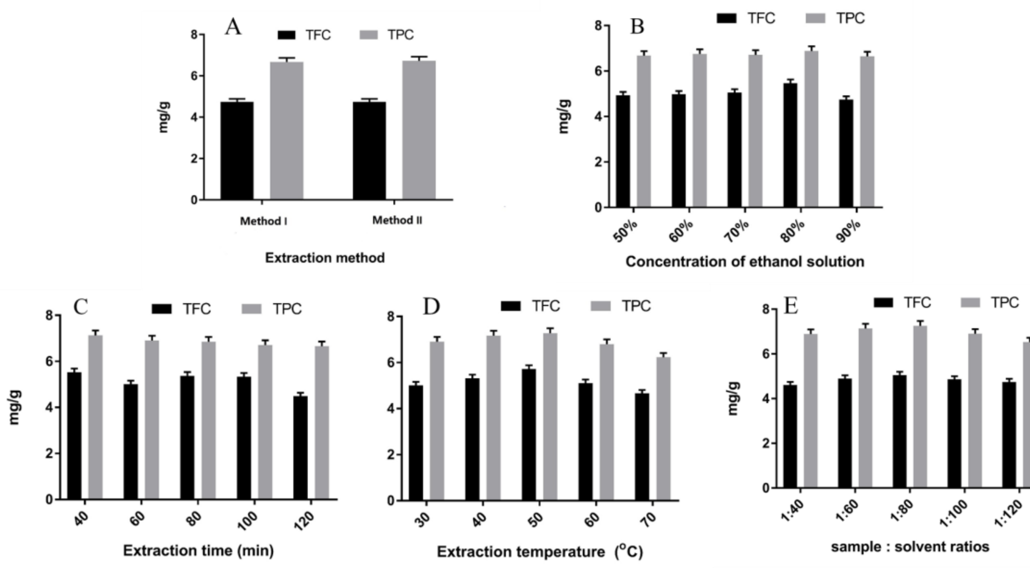

Figure S2. Optimization of samples solutions preparation for the phenolic and flavonoid determination.

A: different extraction methods (method I: firstly, the fruit powder (0.5 g) were extracted by the optimization extraction method of carotenoids, then the extracted residues were dried, and extracted with 30 ml of 80% ethanol solutions for 60 min at 30 °C using ultrasonic bath; method

II: the fruit powder (0.5 g) were soaked with 30 ml of 80% ethanol solutions for 30 min and extracted for 60 min at 30 ^{o}C using ultrasonic bath);

B: different concentrations of ethanol solutions (the fruit powder (0.5 g) were soaked with 30 ml of 50%, 60%, 70, 80% and 90% ethanol solutions, respectively, for 30 min and extracted for 60 min at 30 ^{o}C using ultrasonic bath);

C: different extraction times (the fruit powder (0.5 g) were soaked with 30 ml of 80% ethanol solutions for 30 min and extracted for 40, 60, 80, 100, and 120 min, respectively, at 30 ^{o}C using ultrasonic bath);

D: different extraction temperatures (the fruit powder (0.5 g) were soaked with 30 ml of 80% ethanol solutions for 30 min and extracted for 60 min at 30, 40, 50, 60 and 70 ^{o}C, respectively, using ultrasonic bath);

E: different sample : solvent ratios (the fruit powder (0.5 g) were soaked with 20, 30, 40, 50, 60 and 70 ml of 80% ethanol solutions, respectively, for 30 min and extracted for 60 min at 30 ^{o}C using ultrasonic bath).

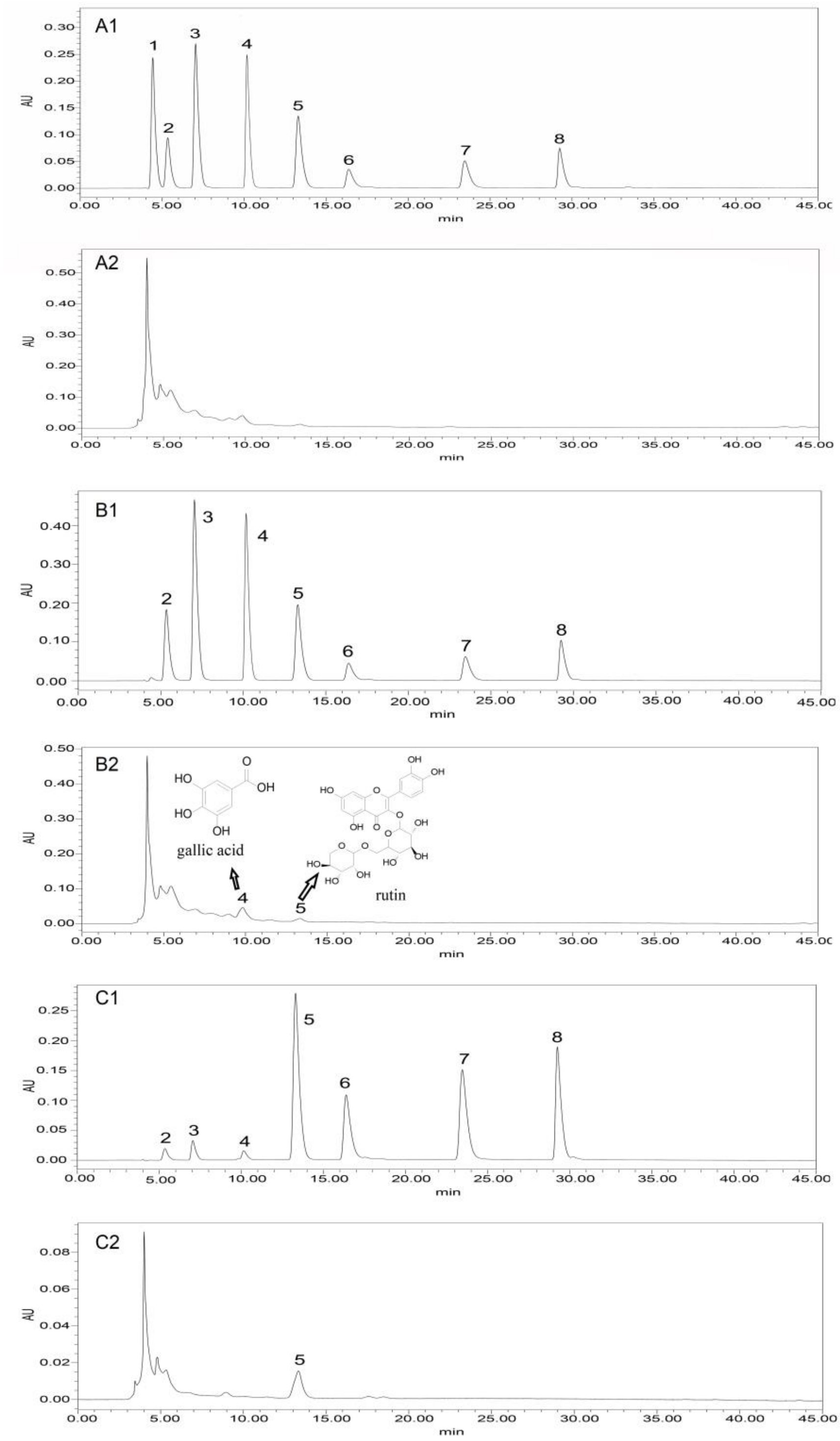

Figure S3. HPLC chromatograms of mixed standard and FLB sample solutions for the determination of phenolic acids and flavonoids. **A1:** mixed standards solution at 280 nm; **A2**: sample solution at 280 nm; **B1**: mixed standards solution at 320 nm; **B2**: sample solution at 320 nm; **C1**: mixed standards solution at 367 nm; **C2**: sample solution at 367 nm; **1.** Gallic acid; **2.** Chlorogenic acid; **3.** Caffeic acid; **4.** Ferulic acid; **5.** Rutin; **6.** Myricetin; **7.** Quercetin; **8.** Kaempferol.

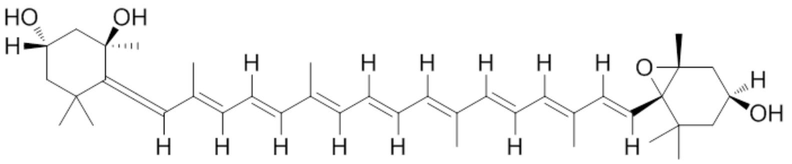

1. neoxanthin

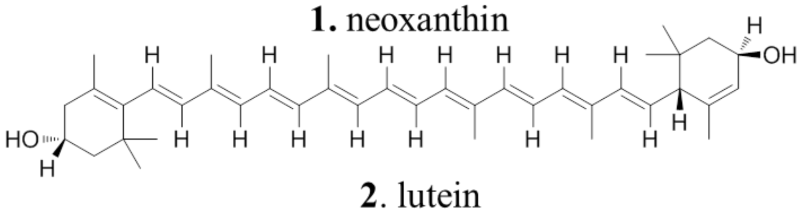

2. lutein

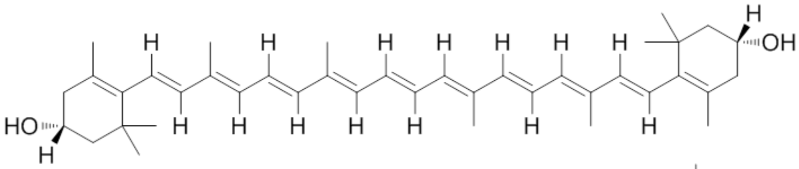

3. zeaxanthin

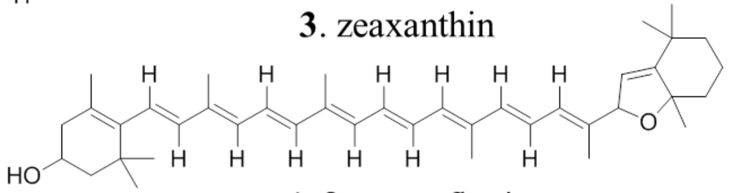

4. β-cryptoflavin

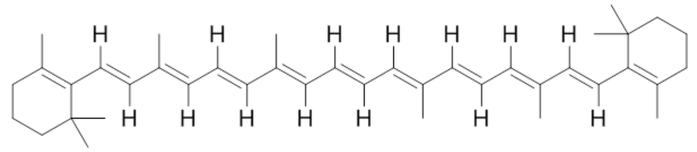

5. β-carotene

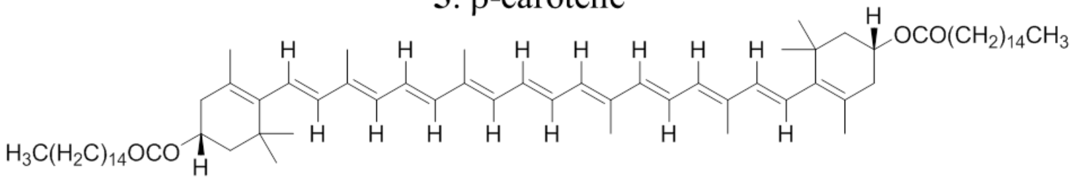

6. zeaxanthin dipalmitate

Figure S4. Chemical structure of the carotenoids analyzed

3 Supplementary Tables

Table S1. Regression equation, correlation coefficients, linearity ranges and limit of detection (LOD) and quantitation (LOQ) of carotenoids

Analytes	Calibration curves	r2	Linear range μg/ml	LOD ng/ml	LOQ ng/ml
neoxanthin	y=165438x+58942	0.9987	0.2500-8.000	59.20	196.1
lutein	y=16207x+15516	0.9963	0.7593-12.15	58.60	194.5
zeaxanthin	y=135260x+306455	0.9959	0.4781-30.60	54.90	192.5
β-carotene	y=38721x+71665	0.9952	1.120-17.92	27.30	90.90
β-cryptoflavin	y=177993x+65285	0.9994	0.3750-3.000	24.20	84.70
zeaxanthin dipalmitate	y=43205x+2753231	0.9992	38.63-618.0	59.50	202.1

Table S2. Precision, repeatability, stability and recovery of carotenoids

Analyte	Precision (RSD, %)		Repeatability	Stability	Recovery (%, n=3)	
	intraday (n=6)	interday (n=6)	(RSD, %, n=6)	(RSD, %, n=6)	mean	RSD,%
neoxanthin	1.64	1.95	-	-	-	-
lutein	1.71	1.87	-	-	-	-
zeaxanthin	1.68	1.86	1.81	1.98	96.2	2.47
β-carotene	1.58	1.61	1.77	1.88	95.7	2.54
β-cryptoflavin	1.66	1.64	1.60	1.64	96.6	2.28
zeaxanthin dipalmitate	1.45	1.65	1.58	1.41	98.4	2.19

Table S3. The results of carotenoids contents (mg/g DW) in fruits of *Lycium barbarum* [a]

No.	zeaxanthin	β-cryptoxanthin	β-carotene	zeaxanthin dipalmitate	TCC	TFC	TPC	TLBP
1	0.0668±0.0017	0.0011±0.0000	0.0651±0.0018	8.534±0.207	20.89±0.60	6.051±0.164	7.937±0.204	32.32±0.80
2	0.0965±0.0025	0.0008±0.0000	0.0699±0.0018	3.936±0.109	13.31±0.34	5.673±0.151	7.945±0.206	36.03±0.96
3	0.0734±0.0018	0.0008±0.0000	0.0326±0.0010	5.574±0.127	14.24±0.41	4.667±0.124	7.823±0.211	29.90±0.64
4	0.0778±0.0016	0.0010±0.0000	0.0680±0.0018	8.505±0.222	21.04±0.61	4.697±0.122	7.173±0.185	38.38±0.10
5	0.0742±0.0018	0.0007±0.0000	0.0958±0.0024	11.25±0.27	23.74±0.69	6.208±0.163	8.972±0.243	26.49±0.58
6	0.0519±0.0013	0.0005±0.0000	0.0627±0.0015	10.562±0.259	21.71±0.62	6.326±0.168	9.960±0.255	37.12±0.99
7	0.0987±0.0024	0.0008±0.0000	0.0443±0.0014	9.881±0.199	18.89±0.55	4.642±0.125	7.821±0.214	31.75±0.88
8	0.1040±0.0028	0.0009±0.0000	0.0347±0.0011	6.808±0.127	14.85±0.43	6.255±0.158	9.056±0.237	26.57±0.57
9	0.1306±0.0034	0.0012±0.0001	0.0591±0.0016	5.867±0.125	13.87±0.40	6.213±0.152	9.060±0.238	25.57±0.54
10	0.0604±0.0015	0.0007±0.0000	0.0328±0.0010	5.995±0.120	16.74±0.48	6.195±0.159	8.095±0.226	22.89±0.49
11	0.1015±0.0028	0.0011±0.0000	0.0715±0.0018	6.413±0.132	17.77±0.53	4.510±0.124	6.992±0.187	33.15±0.89
12	0.1074±0.0026	0.0009±0.0000	0.0538±0.0014	10.03±0.27	24.79±0.70	6.263±0.179	9.045±0.254	34.21±0.99
13	0.0676±0.0017	0.0008±0.0000	0.0793±0.0022	4.125±0.107	12.43±0.32	6.060±0.172	8.739±0.231	40.67±1.12
14	0.0716±0.0017	0.0010±0.0000	0.0605±0.0017	7.292±0.187	16.54±0.44	4.795±0.133	7.707±0.214	25.34±0.56
15	0.1009±0.0025	0.0008±0.0000	0.0930±0.0024	5.852±0.118	14.92±0.42	4.619±0.126	7.471±0.184	30.83±0.78
16	0.0644±0.0016	0.0005±0.0000	0.1252±0.0031	8.546±0.224	23.03±0.64	4.769±0.121	7.601±0.204	45.16±1.15
17	0.0417±0.0011	0.0007±0.0000	0.0713±0.0021	8.640±0.222	22.00±0.63	4.442±0.111	7.526±0.196	42.61±1.10
18	0.0429±0.0012	0.0010±0.0000	0.0526±0.0013	6.821±0.186	17.22±0.49	5.615±0.135	8.578±0.227	39.59±1.09
19	0.0314±0.0006	0.0003±0.0000	0.0572±0.0015	8.554±0.226	19.21±0.54	3.593±0.099	8.184±0.214	38.01±1.02
20	0.0174±0.0004	0.0003±0.0000	0.1086±0.0028	9.726±0.232	22.70±0.62	4.741±0.128	6.891±0.183	38.35±1.06
21	0.0418±0.0011	0.0005±0.0000	0.0621±0.0016	8.721±0.217	20.09±0.58	5.253±0.134	7.338±0.191	32.69±0.79
22	0.0202±0.0004	0.0003±0.0000	0.0530±0.0014	6.204±0.152	16.46±0.44	5.179±0.133	8.413±0.224	27.72±0.61
23	0.0483±0.0012	0.0008±0.0000	0.0732±0.0016	7.785±0.203	17.99±0.52	5.388±0.137	7.286±0.180	30.96±0.77
24	0.0812±0.0021	0.0011±0.0000	0.0881±0.0021	9.932±0.236	21.38±0.63	5.832±0.140	7.499±0.187	26.11±0.51
25	0.0659±0.0018	0.0010±0.0000	0.1328±0.0028	11.58±0.29	25.72±0.72	6.587±0.176	7.880±0.194	26.32±0.52

26	0.1107±0.0035	0.0011±0.0000	0.0900±0.0027	7.957±0.218	21.03±0.61	6.774±0.182	7.624±0.197	36.96±0.99
27	0.1159±0.0035	0.0016±0.0000	0.0688±0.0017	11.50±0.28	26.32±0.73	6.585±0.176	8.045±0.213	36.07±0.96
28	0.1068±0.0031	0.0012±0.0000	0.0676±0.0017	10.63±0.27	24.07±0.70	6.861±0.183	7.956±0.207	29.66±0.55
29	0.1398±0.0038	0.0017±0.0001	0.1219±0.0028	9.426±0.238	22.68±0.64	5.648±0.145	7.599±0.210	37.97±0.99
30	0.1214±0.0034	0.0017±0.0001	0.0903±0.0025	7.102±0.193	22.01±0.61	5.259±0.137	7.019±0.196	29.37±0.56
31	0.1329±0.0038	0.0016±0.0001	0.1276±0.0032	8.243±0.207	23.28±0.65	5.965±0.158	7.609±0.198	28.63±0.52
32	0.0933±0.0024	0.0012±0.0000	0.1157±0.0034	9.024±0.207	17.64±0.50	5.492±0.135	7.687±0.216	28.93±0.60
33	0.0912±0.0023	0.0012±0.0001	0.1452±0.0035	9.497±0.227	21.27±0.61	6.321±0.156	7.608±0.201	26.34±0.49
34	0.1162±0.0032	0.0012±0.0001	0.1920±0.0041	11.91±0.31	28.25±0.81	4.963±0.127	6.694±0.189	28.57±0.52
35	0.0987±0.0028	0.0012±0.0001	0.0971±0.0026	14.68±0.35	29.15±0.82	6.798±0.183	8.076±0.213	38.71±0.10
36	0.1265±0.0035	0.0017±0.0001	0.1296±0.0027	10.46±0.28	25.11±0.73	6.915±0.185	8.377±0.221	29.75±0.63
37	0.0704±0.0018	0.0006±0.0000	0.0864±0.0022	8.435±0.223	21.45±0.63	6.148±0.164	8.627±0.220	30.41±0.69
38	0.0424±0.0011	0.0003±0.0000	0.0822±0.0022	10.84±0.24	20.85±0.61	5.548±0.147	7.761±0.213	30.49±0.70
39	0.0334±0.0010	0.0003±0.0000	0.0679±0.0018	9.764±0.172	21.43±0.61	5.768±0.154	7.510±0.185	28.03±0.59
40	0.0413±0.0011	0.0004±0.0000	0.0259±0.0008	2.707±0.078	9.975±0.250	4.074±0.102	8.096±0.218	37.97±1.03
41	0.0342±0.0009	0.0005±0.0000	0.0334±0.0010	2.926±0.073	9.875±0.246	4.333±0.110	5.703±0.157	26.83±0.58
42	0.0485±0.0013	0.0005±0.0000	0.0561±0.0012	5.517±0.126	15.28±0.43	4.491±0.114	6.751±0.185	25.86±0.49
43	0.0323±0.0008	0.0008±0.0000	0.0648±0.0015	7.650±0.192	19.37±0.57	5.750±0.143	8.493±0.225	26.46±0.49
44	0.0893±0.0024	0.0010±0.0000	0.0793±0.0018	5.886±0.158	14.66±0.41	5.627±0.152	8.282±0.219	37.19±1.00
45	0.0316±0.0007	0.0005±0.0000	0.0293±0.0009	5.120±0.135	14.48±0.42	4.040±0.102	6.923±0.189	25.63±0.49
46	0.0800±0.0018	0.0007±0.0000	0.0914±0.0021	9.304±0.173	21.22±0.61	4.757±0.115	8.012±0.220	27.85±0.57
47	0.0715±0.0017	0.0004±0.0000	0.0290±0.0009	2.529±0.077	10.68±0.31	4.817±0.120	7.712±0.214	25.90±0.47
48	0.0697±0.0017	0.0007±0.0000	0.0849±0.0023	8.442±0.215	16.91±0.49	4.743±0.117	7.019±0.186	37.75±1.03
49	0.0290±0.0009	0.0010±0.0000	0.0427±0.0011	5.409±0.126	14.70±0.40	3.113±0.073	6.983±0.185	25.58±0.57
50	0.0239±0.0008	0.0011±0.0000	0.0593±0.0015	9.837±0.215	16.09±0.43	3.954±0.109	7.170±0.192	20.55±0.46
51	0.0573±0.0014	0.0011±0.0000	0.0497±0.0012	7.860±0.218	18.38±0.53	3.421±0.102	6.623±0.168	24.05±0.53
52	0.0194±0.0008	0.0009±0.0000	0.0657±0.0015	7.924±0.214	19.18±0.53	3.697±0.110	7.0633±0.202	24.30±0.51
53	0.0118±0.0006	0.0010±0.0000	0.0692±0.0018	8.075±0.224	17.45±0.48	3.275±0.083	6.733±0.189	23.38±0.41
54	0.0261±0.0008	0.0011±0.0000	0.0665±0.0016	6.644±0.140	19.73±0.57	4.141±0.114	8.182±0.225	32.14±0.69

55	0.0526±0.0015	0.0013±0.0000	0.0675±0.0018	7.672±0.213	18.12±0.52	4.608±0.112	7.906±0.212	26.87±0.58
56	0.0515±0.0015	0.0012±0.0000	0.1121±0.0026	12.83±0.29	24.70±0.70	3.428±0.098	7.866±0.210	33.62±0.78
57	0.0164±0.0006	0.0011±0.0000	0.0608±0.0018	11.80±0.26	23.65±0.69	3.845±0.103	7.869±0.216	21.69±0.46
58	0.3036±0.0087	0.0032±0.0001	0.1291±0.0028	10.90±0.24	25.13±0.74	4.606±0.115	6.887±0.186	39.00±0.80
59	0.2975±0.0083	0.0034±0.0001	0.1762±0.0042	9.470±0.231	23.16±0.67	4.182±0.105	6.382±0.165	29.44±0.78
60	0.2906±0.0085	0.0029±0.0001	0.1582±0.0043	11.44±0.28	28.71±0.82	5.348±0.134	7.094±0.183	29.26±0.67
61	0.2565±0.0078	0.0028±0.0001	0.1022±0.0025	10.73±0.27	24.58±0.71	5.277±0.124	8.133±0.214	30.36±0.70
62	0.2679±0.0082	0.0032±0.0001	0.1054±0.0026	10.91±0.26	26.09±0.73	5.190±0.132	7.659±0.207	29.89±0.74
63	0.2983±0.0083	0.0033±0.0001	0.1096±0.0026	6.989±0.157	24.41±0.70	4.836±0.122	6.903±0.188	24.15±0.54
64	0.0560±0.0018	0.0005±0.0000	0.0853±0.0021	5.759±0.153	16.18±0.45	5.489±0.125	6.221±0.163	22.52±0.45
65	0.0537±0.0018	0.0004±0.0000	0.0409±0.0012	7.642±0.190	18.24±0.52	5.997±0.132	7.781±0.214	28.52±0.58
66	0.0400±0.0014	0.0004±0.0000	0.0612±0.0015	9.965±0.229	23.00±0.67	6.881±0.156	8.073±0.224	24.46±0.52
67	0.0654±0.0016	0.0006±0.0000	0.0815±0.0018	9.834±0.218	23.07±0.68	5.537±0.136	6.802±0.184	25.04±0.53
68	0.0217±0.0008	0.0005±0.0000	0.0462±0.0012	6.667±0.176	16.96±0.49	3.693±0.108	7.507±0.203	25.31±0.52
69	0.0535±0.0017	0.0004±0.0000	0.0510±0.0012	4.570±0.128	13.02±0.37	3.641±0.092	6.040±0.169	21.46±0.48
70	0.0695±0.0022	0.0006±0.0000	0.0850±0.0023	8.725±0.227	17.38±0.51	4.702±0.124	7.150±0.184	28.73±0.55
71	0.0634±0.0018	0.0001±0.0000	0.0387±0.0012	5.855±0.154	16.98±0.45	4.300±0.109	8.463±0.236	23.38±0.65
72	0.0234±0.0007	0.0003±0.0000	0.0180±0.0006	2.918±0.076	10.51±0.26	2.442±0.072	5.945±0.154	33.43±0.83
73	0.0479±0.0016	0.0001±0.0000	0.0251±0.0009	2.740±0.081	10.45±0.24	2.912±0.073	7.181±0.184	31.30±0.79
74	0.0790±0.0025	0.0001±0.0000	0.0310±0.0010	5.497±0.150	15.84±0.36	4.006±0.110	8.1380±0.214	28.03±0.65
75	0.0306±0.0008	0.0001±0.0000	0.0147±0.0005	6.000±0.108	15.88±0.36	3.350±0.095	7.390±0.193	24.92±0.57
76	0.0682±0.0021	0.0001±0.0000	0.0145±0.0005	2.704±0.078	9.73±0.22	2.828±0.071	7.074±0.187	26.17±0.56
77	0.0120±0.0005	0.0001±0.0000	0.0140±0.0005	3.446±0.097	12.25±0.31	2.676±0.071	6.548±0.164	24.55±0.50
78	0.0191±0.0007	0.0001±0.0000	0.0272±0.0008	4.917±0.146	11.82±0.29	2.900±0.073	7.174±0.185	22.47±0.48

[a] TCC, total carotenoids contents; TFC, total flavonoids contents; TPC, total phenolic contents; TLBP, total polysaccharide contents.

Table S4. The results of antioxidant activity of fruits of *Lycium barbarum*

No.	EC_{50} of DPPH (mg/ml)		EC_{50} of ABTS (mg/ml)		FRAP Fe^{2+}/DW (μmol/g)	
	extract I	extract II	extract I	extract II	extract I	extract II
1	22.96±0.59	2.52±0.06	2.18±0.05	0.41±0.01	17.13±0.41	86.34±2.24
2	28.16±0.69	2.55±0.06	3.46±0.08	0.42±0.01	13.08±0.29	80.90±2.23
3	28.45±0.69	2.68±0.07	4.63±0.12	0.44±0.01	14.52±0.34	86.24±2.24
4	23.96±0.68	2.96±0.07	3.02±0.07	0.50±0.01	14.48±0.35	77.29±2.21
5	24.87±0.69	2.28±0.05	4.63±0.12	0.38±0.01	18.06±0.45	99.34±2.28
6	28.95±0.69	1.93±0.05	4.29±0.11	0.39±0.01	19.26±0.48	99.35±2.28
7	23.76±0.67	2.63±0.06	4.61±0.12	0.44±0.01	18.35±0.45	86.87±2.25
8	22.38±0.66	2.09±0.05	3.21±0.08	0.38±0.01	20.59±0.52	89.38±2.25
9	22.77±0.66	2.06±0.05	3.12±0.07	0.39±0.01	15.64±0.37	94.01±2.27
10	29.33±0.70	2.32±0.05	3.98±0.10	0.42±0.01	14.59±0.34	96.71±2.27
11	23.75±0.66	3.36±0.10	5.21±0.14	0.53±0.02	14.49±0.34	74.69±2.20
12	26.16±0.68	2.14±0.05	4.48±0.11	0.39±0.01	14.67±0.35	98.72±2.28
13	29.25±0.69	2.26±0.05	5.61±0.15	0.40±0.01	13.80±0.31	91.54±2.25
14	28.81±0.68	2.82±0.07	5.57±0.15	0.44±0.01	13.72±0.31	78.53±2.22
15	28.21±0.68	2.89±0.08	4.39±0.11	0.49±0.01	15.02±0.35	82.69±2.23
16	18.13±0.48	2.69±0.06	2.76±0.06	0.45±0.01	13.57±0.32	85.28±2.36
17	20.71±0.49	2.97±0.09	2.61±0.06	0.50±0.01	10.84±0.23	87.51±2.43
18	18.40±0.45	2.30±0.05	2.31±0.05	0.39±0.01	14.18±0.33	94.60±2.64
19	21.46±0.54	2.35±0.05	2.39±0.05	0.47±0.01	12.93±0.29	77.89±2.64
20	25.86±0.64	2.85±0.06	3.29±0.08	0.49±0.01	13.04±0.30	90.00±2.50
21	26.01±0.65	2.41±0.05	3.02±0.07	0.47±0.01	10.53±0.22	75.87±2.08
22	24.73±0.62	2.33±0.05	2.66±0.06	0.46±0.01	10.70±0.22	88.32±2.25
23	25.13±0.65	2.50±0.06	4.01±0.11	0.46±0.01	13.96±0.32	77.69±2.21
24	22.67±0.58	2.35±0.06	4.26±0.11	0.44±0.01	14.30±0.33	86.67±2.24
25	26.58±0.64	2.24±0.05	3.93±0.10	0.43±0.01	12.51±0.28	82.83±2.28
26	19.56±0.47	2.91±0.06	4.12±0.11	0.48±0.01	16.07±0.39	74.42±2.03
27	20.90±0.49	2.49±0.06	4.25±0.11	0.45±0.01	13.37±0.31	79.19±2.18
28	20.79±0.49	2.73±0.07	4.07±0.10	0.47±0.01	15.05±0.35	81.94±2.29
29	20.57±0.47	2.97±0.07	3.93±0.10	0.51±0.01	16.19±0.39	81.10±2.23
30	17.71±0.48	3.14±0.07	3.59±0.08	0.53±0.02	14.93±0.35	84.21±2.33
31	17.77±0.48	2.97±0.07	3.60±0.09	0.49±0.01	15.27±0.36	75.41±2.07
32	21.50±0.58	2.90±0.07	5.10±0.13	0.49±0.01	15.77±0.37	82.71±2.28
33	21.44±0.53	2.97±0.07	4.14±0.10	0.51±0.01	14.21±0.33	91.90±2.57
34	19.14±0.50	3.31±0.08	4.10±0.10	0.57±0.02	15.70±0.37	75.32±2.15
35	18.77±0.48	2.49±0.06	2.46±0.05	0.44±0.01	14.85±0.35	99.70±2.79
36	15.89±0.49	2.48±0.05	4.07±0.10	0.44±0.01	14.75±0.34	87.65±2.43
37	15.20±0.44	3.24±0.07	3.78±0.10	0.44±0.01	17.43±0.42	85.76±2.37
38	16.94±0.48	3.45±0.10	4.56±0.12	0.47±0.01	16.12±0.39	79.39±2.18
39	18.79±0.49	3.83±0.11	3.71±0.10	0.47±0.01	17.61±0.43	76.96±2.11
40	19.90±0.50	2.51±0.06	6.45±0.17	0.49±0.01	13.53±0.31	75.24±2.06
41	20.05±0.50	3.36±0.10	6.59±0.18	0.56±0.02	14.75±0.34	58.98±1.57
42	20.10±0.51	3.23±0.10	6.32±0.17	0.54±0.01	13.43±0.30	73.17±2.00
43	20.26±0.51	2.60±0.06	5.83±0.15	0.46±0.01	15.79±0.37	82.25±2.27
44	19.13±0.50	2.76±0.06	5.54±0.15	0.48±0.01	15.58±0.37	85.97±2.39
45	20.13±0.50	3.63±0.10	5.90±0.16	0.53±0.02	13.04±0.29	66.21±1.79
46	16.04±0.42	2.79±0.07	5.79±0.15	0.49±0.01	16.20±0.39	82.50±2.28

47	20.94±0.52	3.03±0.07	6.83±0.18	0.49±0.01	16.77±0.40	68.98±1.89
48	20.08±0.52	3.12±0.07	5.79±0.15	0.51±0.02	13.49±0.30	75.27±2.06
49	17.45±0.44	2.63±0.05	4.96±0.13	0.50±0.02	14.42±0.33	81.64±2.21
50	21.84±0.55	2.29±0.03	3.38±0.10	0.46±0.01	13.07±0.29	86.84±2.41
51	19.58±0.44	2.75±0.06	3.28±0.09	0.51±0.01	10.97±0.23	80.01±2.21
52	17.83±0.41	2.41±0.05	3.17±0.08	0.48±0.01	11.67±0.25	94.90±2.64
53	23.94±0.68	2.63±0.06	2.76±0.06	0.50±0.01	11.47±0.24	80.09±2.20
54	23.11±0.67	2.16±0.06	2.67±0.06	0.42±0.01	14.24±0.33	98.13±2.74
55	21.75±0.63	2.17±0.04	2.67±0.06	0.43±0.01	12.33±0.27	98.37±2.70
56	19.79±0.48	2.21±0.05	2.00±0.05	0.44±0.01	14.47±0.33	98.21±2.72
57	20.01±0.49	2.22±0.05	2.38±0.06	0.45±0.01	11.29±0.24	98.98±2.77
58	22.31±0.51	2.36±0.05	5.42±0.14	0.44±0.01	10.62±0.22	65.34±1.76
59	22.18±0.52	2.41±0.05	5.01±0.13	0.48±0.02	13.49±0.30	68.96±1.87
60	17.56±0.46	2.03±0.05	6.59±0.18	0.43±0.01	12.46±0.27	78.93±2.17
61	19.78±0.48	1.65±0.03	5.93±0.15	0.41±0.01	12.11±0.26	83.10±2.24
62	22.82±0.53	1.81±0.03	6.62±0.18	0.42±0.01	12.62±0.28	71.50±1.95
63	20.50±0.50	2.33±0.04	6.60±0.17	0.45±0.01	14.27±0.33	83.29±2.30
64	25.45±0.61	2.50±0.05	7.08±0.20	0.45±0.01	10.35±0.21	73.16±1.20
65	22.09±0.57	2.02±0.05	6.10±0.17	0.41±0.01	10.49±0.22	92.15±2.57
66	22.63±0.56	1.72±0.04	7.11±0.19	0.39±0.01	12.36±0.28	93.09±2.58
67	25.29±0.62	2.48±0.05	7.94±0.21	0.41±0.01	12.60±0.28	86.35±2.39
68	21.79±0.53	2.29±0.05	5.91±0.15	0.40±0.01	12.57±0.28	83.92±2.31
69	24.69±0.61	2.62±0.05	6.40±0.17	0.45±0.01	14.92±0.35	69.26±2.00
70	22.17±0.59	2.36±0.05	6.08±0.17	0.41±0.01	11.08±0.23	82.08±2.26
71	21.86±0.54	1.73±0.04	5.00±0.12	0.38±0.01	16.67±0.40	93.55±2.27
72	23.92±0.55	2.76±0.07	5.42±0.15	0.47±0.02	13.27±0.30	78.58±2.22
73	27.32±0.61	2.28±0.058	6.13±0.16	0.41±0.01	12.51±0.28	82.36±2.23
74	21.91±0.51	1.92±0.04	4.83±0.13	0.37±0.01	13.15±0.29	100.34±2.98
75	23.04±0.54	2.24±0.04	4.54±0.11	0.41±0.01	13.89±0.32	82.15±2.27
76	28.34±0.70	2.37±0.05	6.14±0.16	0.42±0.01	11.64±0.25	81.48±2.21
77	25.47±0.65	2.73±0.06	6.31±0.16	0.45±0.02	11.91±0.26	79.30±2.18
78	24.76±0.63	2.33±0.04	5.61±0.15	0.41±0.01	10.78±0.22	84.49±2.23

[a] EC_{50}, the samples concentration for decrease the initial DPPH or ABTS concentration by 50%;

[b] extract I, extraction with hexane/acetone/ethanol (2:1:1, v/v/v); extract II, extraction with 80% ethanol solution.

Table S5. Samples information of fruits of *Lycium barbarum*

Sample no.	Regions	Address of sample collection	Longitude	Latitude	Elevation/m
1	NXZW	Dahe, Hongsibu , Wuzhong , Ningxia	105.97°	37.40°	1272
2	NXZW	Biandangou, Litong , Wuzhong , Ningxia	106.26°	37.78°	1136
3	NXZW	Mingsha , Zhongning , Zhongwei , Ningxia	105.93°	37.46°	1260
4	NXZW	Ning'an , Zhongning , Zhongwei , Ningxia	105.99°	37.27°	1223
5	NXZW	Xinbu , Zhongning , Zhongwei , Ningxia	105.72°	37.48°	1200
6	NXZW	Xinbu , Zhongning , Zhongwei , Ningxia	105.72°	37.47°	1192
7	NXZW	Xinbu , Zhongning , Zhongwei , Ningxia	105.72°	37.47°	1200
8	NXZW	Xinbu , Zhongning , Zhongwei , Ningxia	105.73°	37.47°	1188
9	NXZW	Dazhanchang , Zhongning , Zhongwei , Ningxia	105.68°	37.31°	1174
10	NXZW	Dazhanchang , Zhongning , Zhongwei , Ningxia	105.67°	37.30°	1174
11	NXZW	Dazhanchang , Zhongning , Zhongwei , Ningxia	105.63°	37.38°	1213
12	NXZW	Dazhanchang , Zhongning , Zhongwei , Ningxia	105.63°	37.29°	1312
13	NXZW	Dazhanchang , Zhongning , Zhongwei , Ningxia	105.57°	37.31°	1347
14	NXZW	Zhenluo , Shapotou , Zhongwei , Ningxia	105.43°	37.51°	1179
15	NXZW	Zhenluo , Shapotou , Zhongwei , Ningxia	105.40°	37.54°	1170
16	NXHN	Lihe , Huinong , Shizuishan , Ningxia	106.80°	39.06°	1093
17	NXHN	Yanzidun , Huinong , Shizuishan , Ningxia	106.61°	39.08°	1054
18	NXHN	Yanzidun , Huinong , Shizuishan , Ningxia	106.59°	39.06°	1054
19	NXYC	Nanlaing Farm, Xixia , Yinchuan , Ningxia	106.20°	38.66°	1078
20	NXYC	*Lycium barbarum* Institute, Xixia , Yinchuan , Ningxia	106.14°	38.64°	1074
21	NXYC	Zhenbeibu Towm,Xixia , Yinchuan , Ningxia	106.10°	38.64°	1082
22	NXYC	Zhenbeibu Towm, Xixia , Yinchuan , Ningxia	106.09°	38.64°	1129
23	NXGY	Siying , Yuanzhou , Guyuan , Ningixa	106.16°	36.40°	1496
24	NXGY	Wuying , Yuanzhou , Guyuan , Ningixa	106.16°	36.45°	1525
25	NXGY	Heicheng , Haiyuan , Guyuan , Ningixa	106.10°	36.37°	1517
26	GSBY	Jing'an , Jingyuan , Baiyin , Gansu	105.27°	36.85°	1757

27	GSBY	Wuhe , Jingyuan , Baiyin , Gansu	105.16°	36.93°	1679
28	GSBY	Dongsheng , Jingyuan , Baiyin , Gansu	105.02°	37.00°	1630
29	GSBY	Dongsheng , Jingyuan , Baiyin , Gansu	104.98°	37.02°	1641
30	GSBY	Beitan , Jingyuan , Baiyin , Gansu	104.86°	37.03°	1595
31	GSBY	Beitan , Jingyuan , Baiyin , Gansu	104.87°	37.02°	1595
32	GSBY	Caowotan , Jingyuan , Baiyin , Gansu	104.11°	37.28°	1571
33	GSBY	Dajing , Gulang , Wuwei ,Gansu	103.51°	37.54°	1716
34	GSBY	Dajing , Gulang , Wuwei ,Gansu	103.51°	37.55°	1716
35	GSBY	Dajing , Gulang , Wuwei ,Gansu	103.46°	37.48°	1835
36	GSBY	Xijing , Gulang , Wuwei ,Gansu	103.40°	37.54°	1817
37	GSZY	Jiujiang Forest Farm, Ganzhou , Zhangye , Gansu	100.60°	38.87°	1469
38	GSZY	Jiujiang Forest Farm, Ganzhou , Zhangye , Gansu	100.10°	39.26°	1469
39	GSZY	Banqiao , Linze , Zhangye , Gansu	100.28°	39.31°	1379
40	GSJQ	Dingxin , Jinta , Jiuquan , Gansu	99.49°	40.29°	1128
41	GSJQ	Dingxin , Jinta , Jiuquan , Gansu	99.51°	40.31°	1104
42	GSJQ	Xiba , Jinta , Jiuquan , Gansu	98.73°	40.18°	1189
43	GSJQ	Huahai , Yumen , Jiuquan , Gansu	97.86°	40.21°	1212
44	GSJQ	Chijin , Yumen , Jiuquan , Gansu	97.39°	39.97°	1657
45	GSJQ	Lianghu , Guazhou , Jiuquan , Gansu	96.14°	40.53°	1188
46	GSJQ	Blonge , Guazhou , Jiuquan , Gansu	96.51°	40.53°	1198
47	GSJQ	Blonge , Guazhou , Jiuquan , Gansu	96.51°	40.53°	1198
48	GSJQ	Suoyang , Guazhou , Jiuquan , Gansu	95.71°	40.06°	1534
49	QHGE	Guolemude , Ge'ermu , Hercynian Mongolian Tibetan Autonomous Prefecture, Qinghai	94.78°	36.44°	2754
50	QHGE	Dagler , Ge'ermu , Hercynian Mongolian Tibetan Autonomous Prefecture, Qinghai	95.75°	36.44°	3178
51	QHGE	Nuomuhong Farm, Dulan , Hercynian Mongolian Tibetan Autonomous Prefecture, Qinghai	96.42°	36.45°	2718
52	QHGE	Nuomuhong Farm, Dulan , Hercynian Mongolian Tibetan Autonomous Prefecture, Qinghai	96.40°	36.45°	2696
53	QHDL	Gahai , Delingha , Hercynian Mongolian Tibetan Autonomous Prefecture, Qinghai	97.42°	37.24°	2862
54	QHDL	Tara , Delingha , Hercynian Mongolian Tibetan Autonomous Prefecture, Qinghai	96.74°	37.35°	2817

55	QHDL	Tara , Delingha , Hercynian Mongolian Tibetan Autonomous Prefecture, Qinghai	96.72°	37.32°	2810
56	QHDL	Colucco , Delingha , Hercynian Mongolian Tibetan Autonomous Prefecture, Qinghai	97.16°	37.30°	2815
57	QHDL	Koko , Wulan , Hercynian Mongolian Tibetan Autonomous Prefecture, Qinghai	98.35°	36.96°	2924
58	IMWL	Xianfeng , Urad Front Banner, Bayannur , Inner Mongolia	109.07°	40.63°	1017
59	IMWL	Xianfeng , Urad Front Banner, Bayannur , Inner Mongolia	109.07°	40.62°	1015
60	IMWL	Xianfeng , Urad Front Banner, Bayannur , Inner Mongolia	109.07°	40.61°	1014
61	IMWL	Xianfeng , Urad Front Banner, Bayannur , Inner Mongolia	109.08°	40.60°	1016
62	IMWL	Xianfeng , Urad Front Banner, Bayannur , Inner Mongolia	109.08°	40.63°	1016
63	IMWL	Xianfeng , Urad Front Banner, Bayannur , Inner Mongolia	109.14°	40.59°	1015
64	IMWY	Longxingchang , Wuyuan , Bayannur , Inner Mongolia	108.24°	41.18°	1023
65	IMWY	Longxingchang , Wuyuan , Bayannur , Inner Mongolia	108.29°	41.10°	1023
66	IMWY	Longxingchang , Wuyuan , Bayannur , Inner Mongolia	108.18°	41.17°	1023
67	IMWY	Longxingchang , Wuyuan , Bayannur , Inner Mongolia	108.20°	41.16°	1023
68	IMHJ	Shahai , Hanggin Rear Banner, Bayannur , Inner Mongolia	106.99°	40.96°	1035
69	IMHJ	Shahai , Hanggin Rear Banner, Bayannur , Inner Mongolia	107.00°	40.96°	1035
70	IMHJ	Shahai , Hanggin Rear Banner, Bayannur , Inner Mongolia	106.96°	40.93°	1036
71	XJJH	Tori , Jinghe , Bortala Mongol Autonomous Prefecture, Xinjiang	82.65°	44.54°	332
72	XJJH	Tori , Jinghe , Bortala Mongol Autonomous Prefecture, Xinjiang	82.60°	44.60°	295
73	XJJH	Tori , Jinghe , Bortala Mongol Autonomous Prefecture, Xinjiang	82.70°	44.56°	308
74	XJJH	Tori , Jinghe , Bortala Mongol Autonomous Prefecture, Xinjiang	82.63°	44.62°	301
75	XJJH	Tori , Jinghe , Bortala Mongol Autonomous Prefecture, Xinjiang	82.68°	44.56°	312
76	XJJH	Tori , Jinghe , Bortala Mongol Autonomous Prefecture, Xinjiang	82.65°	44.57°	305
77	XJJH	Tori , Jinghe , Bortala Mongol Autonomous Prefecture, Xinjiang	82.62°	44.58°	303
78	XJJH	Tori , Jinghe , Bortala Mongol Autonomous Prefecture, Xinjiang	82.64°	44.59°	293
